# The role of WT1 in breast cancer: clinical implications, biological effects and molecular mechanism

**DOI:** 10.7150/ijbs.39958

**Published:** 2020-02-24

**Authors:** Ye Zhang, Wen-Ting Yan, Ze-Yu Yang, Yan-Ling Li, Xuan-Ni Tan, Jun Jiang, Yi Zhang, Xiao-Wei Qi

**Affiliations:** 1 Breast and Thyroid Surgery, Southwest Hospital, Army Medical University, Chongqing 400038, China; 2 Breast and Thyroid Surgery, Chongqing General Hospital, University of Chinese Academy of Sciences, Chongqing 400013, China

**Keywords:** WT1, breast neoplasms, prognosis, carcinogenesis

## Abstract

Although Wilms' tumor gene 1 (WT1) was first cloned and identified as a tumor suppressor gene in nephroblastoma, subsequent studies have demonstrated that it can also play an oncogenic role in leukemia and various solid tumors. WT1 exerts biological functions with high tissue- and cell-specificity. This article reviews the relationship between WT1 and breast cancer from two aspects: (1) clinical application of WT1, including the relationship between expression of WT1 and prognosis of breast cancer patients, and its effectiveness as a target for comprehensive therapy of breast cancer; (2) the biological effects and molecular mechanisms of WT1 in the development and progression of breast cancer, including proliferation, apoptosis, invasion, and metastasis of breast cancer cells.

## Introduction

Malignant tumor is the second most common cause of death worldwide. There are estimated 18.1 million new cases of malignant tumors and 9.6 million malignant tumor-related deaths in 2018 1. In China, the incidence of malignant tumor is about 200/100,000, with an increasing trend, and deaths caused by malignant tumors account for nearly 20% of total deaths 2. The biological events such as occurrence, invasion, and metastasis of malignant tumors are regulated at multiple levels and by various factors. One of the key factors is the imbalance between oncogenes and tumor suppressor genes. It is necessary to explore new functions of existing oncogenes or tumor suppressor genes in order to clarify the mechanism of malignant tumor occurrence and progression, which then will benefit to find effective diagnostic and therapeutic targets 3.

Wilms' tumor 1 (*WT1*) was originally cloned and identified as a tumor suppressor gene in Wilms tumors 4. Then, further studies have shown that WT1 can regulate a series of target genes and signaling pathways in the formation of organs such as the heart, kidney, spleen, and retina 5-9. Moreover, the wild-type *WT1* was detected in leukemia and solid tumors, suggesting its role as an oncogene 10, 11. This article focuses on the relationship between *WT1* and breast cancer to comprehensively understand the effects and the underlying mechanisms of *WT1* in breast cancer.

## Structure of* WT1* gene, mRNA, and protein

**Figure 1:**
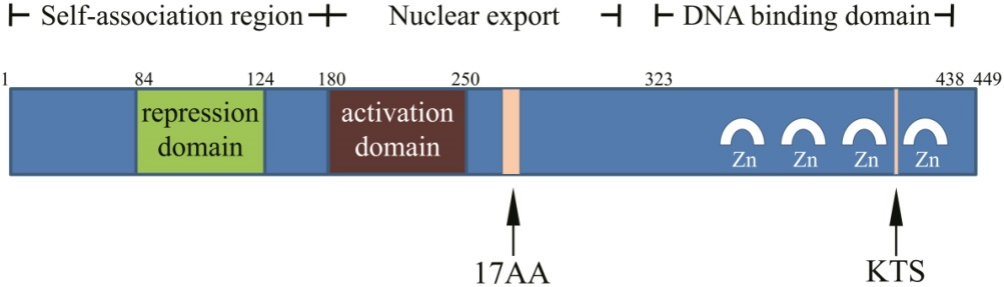
Structure of WT1 protein.

*WT1* was originally identified as a tumor suppressor gene in nephroblastoma, and was inactivated in tumors. The population with *WT1* gene mutation was more susceptible to nephroblastoma 4, 12. *WT1* is located on short band 1 region 3 of human chromosome 11 and is about 50 kb in length with 10 exons, and is rich in GC homologous sequences 13. *WT1* has two splice sites: a 51-bp long one between the exon 5 zinc finger structure and the glutamic/ proline amino acid (AA)-rich region encodes 17 AA; a 9-bp long one between the third and fourth zinc finger structures at the end of the exon 9 encodes three AA (lysine-threonine-serine, KTS) (Figure 1). The splicing at two sites of *WT1* mRNA could produce four major isoforms: WT1 A (17AA-/KTS-), WT1 B (17AA-/ KTS+), WT1 C (17AA+/KTS-), and WT1 D (17AA+/ KTS+) 13-17. Since no physiological function of WT1 C has been reported in literature, it was removed from the National Center for Biotechnology Information (NCBI) Entrez Gene database in April 2011, and another two isomers WT1 E and WT1 F were added. The corresponding RefSeq NM (mRNA) and NP (protein) numbers for WT1 A, WT1 B, WT1 D, WT1 E, and WT1 F are as follow: NM_000378.4 → NP_000369.3, NM_024424.3 → NP_077742.2, NM_024426.4 → NP_077744.3, NM_001198551.1 → NP_001185480.1, and NM_001198552.1 → NP_001185481.1, respectively 18.

The full length product encoded by *WT1* is a 52-54-kD protein. The C-terminal consists of four zinc finger structures, each of which has two cysteines and two histidines. The zinc finger domain is associated with DNA binding, regulating gene transcription. Proline, glutamic acid, serine, and glycine are abundant in the N-terminal, and it can interact with DNA and RNA and participate in protein interaction. KTS insertion or deletion can alter the specific combination of WT1 protein and DNA, and the WT1 (KTS-) isomer acts as a transcription factor 13-17.

## Significance of WT1 in embryogenesis

During embryogenesis, WT1 participates in the formation of organs such as the heart, kidney, spleen, and retina by regulating multiple target genes and signaling pathways 5-9. The low expression of *WT1* is observed in normal tissues because of limited expression sites, while only microexpression of *WT1* is reported in glomerular podocytes, granulosa cells, the endometrium, testicular cells, and immature hematopoietic cells in the spleen and bone marrow 5, 6, 19, 20. This indicates that *WT1* expression is cell- and tissue-specific.

During embryonic development, WT1 affects the transcriptional expression of genes by regulating the promoter activity of various growth factors and their receptors. The known target sequence of WT1 is 5′-GCGGGGGCG-3′, and the target genes including insulin-like growth factors (IGFs) and their receptor (IGF1R), platelet-derived growth factor A (PDGFA), epidermal growth factor (EGF), transforming growth factor beta (TGF-β), macrophage colony-stimulating factor (M-CSF), multidrug resistance 1 (MDR1), Bcl-2, c-MYC, human telomerase reverse transcriptase (hTERT), and cyclin E. WT1 protein can also bind to GC-rich homologous sequence of *WT1* gene to regulate its own expression. The effect of WT1 on its target genes (activation or inhibition of transcription) is highly cell- and tissue-specific, and is also affected by isomers and interactions with other genes 10, 11, 13-17. Martínez-Estrada et al.^21^ found that WT1 can participate in embryonic heart development by inducing epithelial-mesenchymal transition (EMT). Knockout of *WT1* could reduced the number of mesenchymal progenitor cells and inhibited cell differentiation, upregulating E-cadherin and downregulating N-cadherin, alpha smooth muscle actin, and Snail. WT1 can inhibit E-cadherin (*CDH1*) transcription and promote *SNAIL* transcription by binding to their promoters. Kirschner et al. 22 revealed that WT1 can transcribe vascular genes, such as cadherin 5 (*CDH5*, vascular cadherin), vascular endothelial growth factor and angiopoietin, and activate their expression. Based on these findings, it is concluded that WT1 can promote the transcription of vascular endothelial genes and participate in blood vessel.

## Role of *WT1* in breast cancer

As mentioned above, with the research progressed, the function of *WT1* beyond as a tumor suppressor factor has been reported. Therefore, it is generally believed that *WT1* is inactivated by point mutation or deletion so as to trigger malignant tumor. *WT1* inactivation results in the loss of specific target gene binding, interfering with normal regulation of transcription, cell growth, differentiation, and proliferation, and eventually leads to tumorgenesis. However, it is also found that *WT1* can be detected in various tumors, and mutation analysis has suggested that *WT1 rarely* mutates, indicating that *WT1* could also function as an oncogene. WT1 may promote cell proliferation and participate in tumor cell infiltration and metastasis by regulating the transcriptional expression of growth factor genes.

Recent findings provide new evidence for the oncogene function of *WT1* 23, 24. Harkamp et al. 23 suggested that the HtrA2 (HtrA serine peptidase 2) is the binding partner of WT1. Inhibiting HtrA2 activity can block WT1 proteolysis, suggesting that HtrA2/OMI can degrade WT1. Under apoptosis-inducing conditions, HtrA2/OMI-dependent WT1 proteolysis leads to the loss of regulation of the downstream target genes of WT1 and increased apoptosis. Vicent et al 25 found that RNA interference (RNAi) of WT1 significantly reduced tumor burden, induced cell senescence, and inhibited tumor cell proliferation in a proto-oncogene *KRAS*-induced tumor model. WT1 also affects cytoskeletal rearrangement and participates in cell movement by regulating actin activity, which indicated the possible role of WT1 in cancer cell invasion and migration 26.

### *WT1* expression in breast cancer

In 1997, Silberstein et al 27 immunohistochemically analyzed invasive breast cancer tissues from 21 patients and found that WT1 was expressed in normal breast ducts and lobules, but scarce in breast cancer cells. Based on the aforementioned results, *WT1* was regarded as a tumor suppressor gene in breast cancer. In 1999, Loeb et al 28 used reverse transcription (RT)-PCR to detect the expression of *WT1* mRNA in breast cancer tissues of 31 cases, and found that *WT1* expression was high in primary breast cancer (27/31) and low in normal breast tissue (1/20), suggesting that *WT1* may act as an oncogene. Oji et al 29 sequenced the DNA of 36 breast cancer tissues and confirmed that the *WT1* detected in the above breast cancer samples was not mutated. Therefore, the results confirm that wild-type *WT1* acts as an oncogene in breast cancer. Furthermore, Provenzano et al. 30 compared the expression of 6 proteins in 266 non-small cell lung cancer samples and 837 primary breast cancer samples, and results showed that cytoplasmic staining for WT1 favoured breast origin. In addition, Craig et al. 31 detected focal amplifications of WT1 in triple-negative breast cancer (TNBC). We have previously demonstrated that single-nucleotide polymorphisms (SNPs) of WT1 can decrease *WT1* mRNA expression and are associated with reduced breast cancer risk 32. Recently, a histopathological study confirmed that WT1 protein expression is higher in breast tumor than in tumor-free tissue 33.

### Relationship between *WT1* and prognosis in breast cancer

Miyoshi et al. 34 detected the expression of *WT1* mRNA in 99 breast cancer cases. The results suggested that *WT1* expression was not associated with patients' clinicopathological parameters, but patients with high *WT1* expression had significantly lower overall survival (OS) than patients with low WT1 expression. Besides, multivariate analysis showed that *WT1* was an independent indicator of poor prognosis for breast cancer patients. We found that high *WT1* mRNA expression was associated with high histological grade, estrogen receptor (ER)-negative status, basal-like subtype, and ERBB2 (erb-b2 receptor tyrosine kinase 2, HER2) subtype in breast cancer patients. Patients with high WT1 expression had a shorter disease-free survival (DFS) than patients with low WT1 expression 35. Choi et al. 36 performed an immunohistochemical analysis of 100 invasive ductal breast cancer cases and showed that WT1 expression was closely related to tumor grading and DFS. Patients with positive WT1 expression had significantly longer DFS than patients with negative WT1 expression. Our meta-analysis showed that WT1 expression are not significantly related to OS and DFS of breast cancer patients, and the variance among studies may be due to different detection methods and antibody inconsistency 37.

### Relationship between *WT1* and its potential application in breast cancer

Higgins et al. 38 studied the safety and efficacy of WT1 immunotherapy in combination with standard neoadjuvant therapy in patients with positive WT1 expression. This immunotherapy induces T cell recognition of tumor antigens by vaccination, and activates the immune response to produce tumor antibodies. Results showed patients treated by aromatase inhibitors were well tolerated to concurrent therapy and more likely to produce WT1-specific antibodies, whereas patients who received neoadjuvant chemotherapy or trastuzumab had relatively less WT1-specific antibodies. In another study, WT1 peptide-pulsed DC vaccination was used to treat advanced cancer patients. Results showed that all the four patients with advanced breast cancer achieved clinical response 39. Li et al 40 suggested that the *WT1* SNP rs1799937 is associated with pathological complete response (pCR) after anthracycline and/or taxane based neoadjuvant chemotherapy and that patients with the TT genotype are more likely to achieve pCR. In addition, Wu et al. 41 reported that among 389,795 *WT1* SNPs associated with pCR, rs6044100 and rs1799937 were significantly associated with pCR after neoadjuvant chemotherapy, suggesting that WT1 may be a potential target in breast cancer.

### *WT1* and breast cancer cell proliferation and apoptosis

In 2002, Zapata-Benavides et al. 42 found that stimulating breast cancer cell proliferation with 17β-estradiol significantly increased WT1 protein expression, while, tamoxifen or all-trans retinoic acid could inhibit cell proliferation. Moreover, interfering with WT1 expression inhibited cell proliferation and decreased cyclin D1 expression. Caldon et al. 43 also reported that interfering with WT1 expression decreased the proportion of cells in S phase, leading to cell proliferation arrest, differentiation, and apoptosis, which may be related to decreased expression of cyclin D1. Nasomyon et al. 44 found WT1 induced estrogen-independent growth and anti-estrogen resistance in MCF7 cells through enhanced expression of EGFR and HER2. Navakanit et al. 45 found that silencing WT1 could inhibit the growth of MCF-7 cell line. In addition, Tuna et al. 46 found that HER2 could upregulate WT1 expression through the Akt signaling pathway, promoting breast cancer cell proliferation and inhibiting apoptosis, suggesting that WT1 can promote breast cancer cell proliferation. As HER2 plays an important role in the stemness maintain of breast cancer stem cells, WT1 may play a role in maintaining the biological characteristics of breast cancer stem cells.

WT1 can increase the malignancy of breast cancer, leading to poor prognosis in patients with high WT1 expression. Han et al. 47 reported that *WT1* overexpression antagonized tamoxifen-induced apoptosis in MCF-7 cells, and further studies showed that WT1 could bind to the ERα promoter and inhibit its transcriptional activity, which finally led to ERα downregulation and caused estrogen resistance. Furthermore, Wang et al. 48 found that WT1-upregulated EGFR (epidermal growth factor receptor, HER-1) expression was associated with endocrine therapy resistance in breast cancer.

The major WT1 subtypes have inhibitory functions, e.g., WT1 (17AA+/KTS+), WT1 (17AA+/KTS-), and WT1 (17AA-/KTS+) can inhibit caspase-3, caspase-9, and Bax expression to play an anti-apoptotic role 49. Graidist et al. 50 found that the expression of two short WT1 subtypes, i.e., T-KTS+ and T-KTS-, and two major subtypes, i.e., WT1 (17AA+/KTS+) and WT1 (17AA+/KTS-), can inhibit the expression of the apoptotic genes such as p53, Bak, and caspase-7 in MCF-7 cells, inhibiting doxorubicin-induced cell death. Navakanit et al. 45 found that after RNAi downregulated WT1 in MCF-7 cells, doxorubicin toxicity was significantly increased, causing increased apoptosis of cells deaths.

Much of the current research supports the premise that WT1 plays an oncogenic role in breast cancer, promoting cell proliferation and inhibiting apoptosis, but some other studies have different findings. For example, Zhang et al. 51 reported that WT1 inhibited breast cancer cell growth by regulating the stability of β-catenin. Reizner et al. 52 found that WT1 interacted with ERα and blocked the transactivation of ERα to the IGF-1R, resulting in the inhibition of IGF1R transcription and decreased cell proliferation. Wang et al. 53 showed that WT1 inhibited the malignant transformation of the MCF10A normal breast cell line and its tumorigenic ability in nude mice. Burwell et al. 54 transduced the WT1 (exon 5 [Ex5]-/KTS-) and WT1 (Ex5+/KTS+) isoforms into H16N2 cells and found that WT1 (Ex5-/KTS-) increased p21 expression and cell proliferation with declining reproductive ability and G2 arrest. However, WT1 (Ex5+/KTS+) did not affect p21 expression, but promoted EMT, which was demonstrated by membrane E-cadherin translocation into the nucleus, and cells developing mesenchymal cell-like morphology. The study by Wang et al 53 showed that high WT1 expression inhibited MCF10AT3B cell growth and inhibited their tumorigenic ability in nude mice. Jomgeow et al. 55 found that WT1 (17AA-/KTS-) expression induced three-dimensional morphological changes in the ZR-75 and SK-BR-3 breast cancer cell lines, and significantly reduced the sizes of both cell types. These results suggest a complex role of WT1, which differs between cell types and even in different subgroups of similar cells. WT1 has multiple isomers, and their function depends on the ratios and roles, which should be further clarified. Recently, Lee et al. 56 found that cugWT1 (*WT1* translated from a CUG site) and augWT1 (*WT1* translated from an AUG site) have different effect in breast cancer because they have different promoters of protein synthesis. cugWT1 is highly expressed in breast cancer cells, which can mediate tumor cell transformation and up-regulate c-*MYC*, *BCL2*, and *EGFR* expression; silencing it leads to decreased anchorage-independent growth and proliferation of breast cancer cells. augWT1 usually functions as a tumor suppressor gene, and cugWT1 overexpression inhibits tumor cell colony formation and inhibits expression of the above genes by recruiting histone deacetylase (HDAC1).

### *WT1* and breast cancer cell invasion and migration

Zhang et al. 57 found that WT1 is involved in breast cancer cell migration mediated by focal adhesion kinase and keratinocyte growth factor (KGF). Artibani et al. 58 reported that knocking out WT1 in the TNBC MDA-MB-157 cell line significantly increased cell movement, invasion, and metastasis, but had no effect on cell proliferation and apoptosis, and tumor stem cell characteristics. Bissanum et al. 59 suggested that, in TNBC, WT1 B and WT1 C upregulate EphA2 (EPH receptor A2) expression, activate β-catenin signaling transduction, and promote vimentin expression, enhancing cell migration and promoting the formation of vasculogenic mimicry. Our study showed that TGF-β could upregulate WT1 in breast cancer cells, altering cell epithelial morphology to mesenchymal morphology and promoting the proliferation, invasion, and migration of cancer cells significantly 60.

### Regulatory factors of the *WT1* gene in breast cancer cells

As a transcription factor, WT1 can widely regulate a variety of target genes 61. Chesor et al. 62 found that silencing WT1 in MDA-MB-468 cells significantly inhibited the expression of PDGF receptor alpha (PDGFRA, involved in cell proliferation) and guanine nucleotides exchange factor 1, which is involved in migration. Keating et al. 63 believed that *WT1* is a tumor suppressor gene and can reduce thromboxane receptor α expression by inhibiting its transcription.

At present, there are few studies on the upstream regulatory factors of *WT1* expression. Tuna et al. 64 found that IGF1 increased WT1 protein expression in a time-dependent manner through the Akt pathway in MCF-7 cells. In addition, the *HER2*/neu gene regulated WT1 protein expression through Akt 46. Xie et al. 65 found that *WT1* was highly expressed in breast cancer tissues and microRNA-193a could inhibit its expression by binding to the *WT1* coding region. MicroRNA-193a expression in breast cancer was negatively correlated with WT1 expression, and overexpression of WT1 could partially offset the activity of microRNA-193a. Cheng et al. 66 reported that WT1, a transcriptional inhibitor of STIM1 (stromal interaction molecule 1), was upregulated after TGF-β treatment in MDA-MB-231 cells, and *WT1* knockdown partially reversed the STIM1 downregulation caused by TGF-β. Our previous study showed that TGF-β can induce EMT by upregulating WT1 breast cancer cells, resulting in promoting proliferation, invasion, and migration of tumor cells 60.

## Conclusion

WT1 and its encoded protein are highly expressed in hematological malignancies and solid tumors such as breast cancer, lung cancer, pancreatic cancer, and prostate cancer. In addition, patients with WT1 gene amplification could produce antibodies against WT1 protein and cytotoxic T lymphocytes, indicating that WT1 protein is highly immunogenic and could serve as a therapeutic target. Currently, WT1 vaccines against leukemia, breast cancer, lung cancer, and pancreatic cancer are undergoing phase I or II clinical trials, and their efficacy and adverse reactions are also being under investigation. It is important to determine whether WT1 plays a significant role in the invasion, migration, and metastasis of breast cancer and explore its molecular mechanism. Clinical application of WT1for the treatment of breast cancer still needs to be investigated by more well-designed clinical trials.

## Figures and Tables

**Figure 2:**
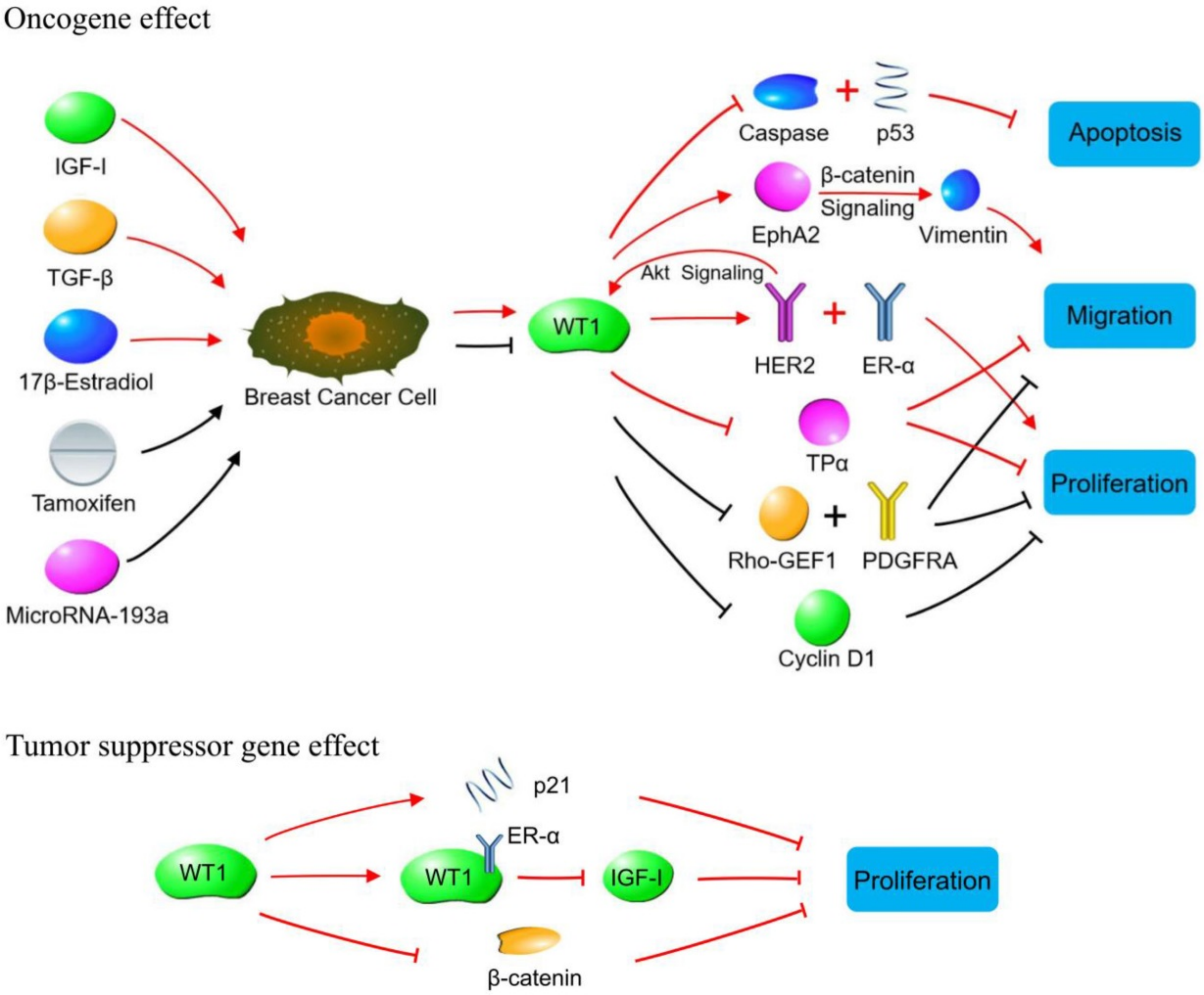
WT1 plays a role in promoting or suppressing cancer cells through various signaling pathways in breast cancer. Oncogene effect: Red lines indicate signaling pathways initiated by increasing WT1 expression. The WT1 expression is up-regulated by IGF-1, TGF-β and 17β-estradiol in breast cancer cells. The up-regulated WT1 can regulate the proliferation, migration, and apoptosis of breast cancer cells by acting on molecules or signaling pathways such as Caspase family, p53, EphA2, HER2, ER-α, and TPα. Black lines indicate the biological effects produced by inhibiting WT1 expression. Tamoxifen and MicroRNA-93a inhibit the expression of WT1 in breast cancer cells, so that the expression of Rho-GEF1, PDGFRA and cyclin D1 is inhibited, leading to the suppressed proliferation of breast cancer cells. Tumor suppressor gene effect: WT1 inhibits breast cancer cells by binding to ER-α, or promoting p21 expression, or inhibiting β-catenin.
